# The relationship between challenge-hindrance stressors and innovative behavior among medical postgraduates in China: the mediation role of academic engagement and the moderating effect of relaxation

**DOI:** 10.1080/10872981.2024.2379110

**Published:** 2024-07-17

**Authors:** Dan Bao, Faridah Mydin, Shahlan Surat, Yanhong Lyu, Dongsheng Pan, Yahua Cheng

**Affiliations:** a Faculty of Education, Universiti Kebangsaan Malaysia, Bangi, Selangor, Malaysia; b Department of Humanities and Social Sciences, Hubei University of Medicine, Shiyan, Hubei, China; c Department of Obstetrics and Gynecology, Xijing Hospital, Fourth Military Medical University, Xi’an, China; d Department of Orthopaedics, Xijing Hospital, Fourth Military Medical University, Xi’an, China; e School of Government, Shanghai University of Political Science and Law, Shanghai, China

**Keywords:** Challenge stressors, hindrance stressors, academic engagement, innovative behavior, relaxation, medical postgraduates

## Abstract

This study investigated the relationship between challenge-hindrance stressors and innovative behavior of medical postgraduates in China, examining the mediating role of academic engagement and the moderating effect of relaxation. Drawing from a sample of 437 medical postgraduates from three Chinese universities, our findings revealed that challenge stressors positively correlated with innovative behavior, while the direct relationship between hindrance stressors and innovative behavior was not statistically significant. Furthermore, academic engagement mediated the relationship between two types of stressors and innovative behavior. Challenge stressors enhanced academic engagement, which in turn fostered innovative behavior. Conversely, hindrance stressors were found to diminish academic engagement, which in turn indirectly limited innovative behavior. Additionally, relaxation was identified as a moderating factor that helped mitigate the negative effects of hindrance stressors on academic engagement and indirectly on innovative behavior. These results suggested that academic engagement as a mechanism played a pivotal role in determining how different stressors influenced innovative behavior, underscoring the need for stress management, particularly through relaxation techniques, to maintain high levels of academic engagement and innovative behavior. This study offers practical insights for medical education policymakers and educators in China, emphasizing the importance of balancing stressors and incorporating relaxation practices to enhance the innovative capabilities of medical postgraduates in demanding academic environments.

## Introduction

With the robust advancement of technological revolution and the escalating demands for healthcare services, innovation has become a vital lifeline for the reform and development of medical education in China [1,2]. Medical postgraduates, trained as the nation’s high-level medical talents, serve as a pivotal force leading the production of medical knowledge and technological innovation in China. In this evolving landscape, the innovative abilities of medical postgraduates become crucial, equipping them to adeptly navigate the rapid advancements in technological innovation and the escalating demands for healthcare services. Innovative behavior refers to the process wherein an individual within a specific domain or environment, adopts novel and unique modes of thinking, proposes fresh perspectives or ideas, and subsequently translates them into tangible actions [3–5]. The relevant literature on innovative behavior indicates that it not only enhances the individual’s capability to seize external opportunities and adapt to the external environment, thereby facilitating personal growth, but also boosts their future professional performance and competitiveness [4,6,7].

Given the demanding nature and complexity of medical specialties, medical postgraduates face the formidable task of absorbing extensive knowledge, participating in diverse medical research activities, and dedicating significant effort within the constraints of limited time and energy [8–10]. As a result, the strenuous requirements of both learning and research can lead to stress [11,12]. Related studies showed that medical students are subjected to the pressure of high demands experience study fatigue, depression and burnout [13–15]. But if we focus solely on reducing stress, can we achieve the desired results? In addition, previous studies in medical education have mostly focused on the negative effects of stress, believing that stress can undermine students’ enthusiasm and initiative at study [9,12]. Since Cavanaugh introduced the concept of Challenge-Hindrance Stressor Framework, the positive effects triggered by stressors have gradually gained attention from scholars [16]. For the medical postgraduates, although hindrance stressors tend to result in negative consequences, challenge stressors may lead to positive attitude and behavioral outcomes. However, there is a dearth of studies that apply the challenge -hindrance stressor framework to educational environments, especially in medical education. A research gap exists in exploring the distinct roles of challenge-hindrance stressors and understanding how they influence the mechanisms of innovative behavior among medical postgraduates. To fill the theoretical and empirical gaps, this study aims to investigate the mechanisms through which different stressors affect innovative behavior among medical postgraduates in China, and identify the moderating factor influenced these relationships.

## Challenge-hindrance stressors and innovative behavior

The basic premise of the Challenge-Hindrance Stressor Framework is that stressors can be conceptualized into the two broad categories of challenge stressors and hindrance Stressors. Challenge stressors are perceived as opportunities for growth, learning, and skill development. They typically involve tasks or situations that are demanding but provide individuals with a sense of control and mastery, while hindrance stressors that are beyond individuals’ control and impede personal growth and achievement [16,17]. In the experience of medical postgraduates, challenge stressors are associated with heavy academic workload, time pressure, task complexity, and a sense of responsibility, etc.; in contrast, hindrance stressors involve interpersonal conflicts, study or research obstacles, limited resources and a sense of insecurity, etc. Previous studied in work environments demonstrated the positive correlation between challenge stressors and desirable work outcomes, as opposed to the negative outcomes associated with hindrance stressors [17–22]. Therefore, if there are significant differences in the source of stressors, the relationship between them and innovative behavior of medical postgraduates may also vary.

According to the definition of innovative behavior from Scott and Bruce [3], the innovative behavior of medical postgraduates typically involves the actions taken by students during their learning and research experiences, where they continuously create and develop new knowledge, skills, and methods through the discovery, analysis, and resolution of problems. According to the Expectancy Theory [23], overcoming challenge stressors can enhance individual performance and potential, leading to higher rewards. Therefore, individuals adopt proactive strategies towards this type of stressors, fully mobilizing their subjective initiative, making it easier to generate innovative ideas. On the other hand, hindrance stressors obstruct goal achievement, accompanied by reduced rewards. As a result, individuals develop negative expectations towards this type of stressors, leading to conformity and making it difficult for innovative ideas to emerge. Secondly, based on the Job Demands-Control Model [24], when faced with challenge stressors, individuals can utilize their own knowledge or effort to enhance control over their study, and being motivated in this way increases proactive behavior. Conversely, when faced with hindrance stressors, individuals are unable to strengthen their control over study by changing themselves, leading to high internal tension, diminished confidence, and a reduction in proactive behavior. Finally, draw upon the Conservation of Resources Theory [25], individuals tend to invest time, energy, and other resources in efforts to overcome stress that brings high rewards, promoting the appreciation of their own resources. This positive investment of resources is more likely to result in creative outcomes, such as new methods and technologies. In sum, challenge stressors may motivate medical postgraduates to actively learn new methods, explore new topics, and thereby promotes the generation of innovation behavior, while hindrance stressors may lead them conserve their energy and reduce their investment in learning or research work, adopting a passive approach, choosing the simplest and most conventional solutions to problems, thereby reducing the likelihood of generating innovative behavior. Therefore, we speculate that:

H1: Challenge stressors are positively related to innovative behavior.

H2: Hindrance stressors are negatively related to innovative behavior.

## Academic engagement as a mediator

Existing literature has found that the mechanism by which challenge and hindrance stressors impact innovative behavior often involves various mediating effects. Some studies have pointed out that work engagement is an outcome of stressors, suggesting that individuals are more inclined to actively invest in their learning and work when they believe their exceptional efforts will lead to higher achievements [19,26]. Additionally, a study has indicated that work engagement plays a complete mediating role between challenge-hindrance stressors and individual creativity [27]. Similar to work engagement, academic engagement is characterized by vigor, dedication, and absorption. It not only represents a positive and fulfilling state of mind related to study but also encompasses positive psychological traits like optimism and resilience [28]. These traits are critical for the holistic development of medical postgraduates, influencing their learning initiatives, creativity and broader professional development [29,30]. When medical postgraduates assess different stressors they are facing, the result of this assessment determines their attitude and behavior in addressing the stress. According to the Self-Determination Theory, when individuals face challenges, their inherent potential drives them to choose tasks that are conducive to their personal development, leading to higher levels of academic engagement from challenge stressors. Conversely, when confronted with hindrance stressors, the situation is quite the opposite [31]. Empirical studies have also demonstrated that an individual’s state of academic engagement is associated with higher levels of academic performance and enhances psychological well-being [32,33]. Greater engagement in tasks is favorable for fostering innovative behavior in individuals [34]. Therefore, we speculate that:

H3: Challenge stressors are positively related to academic engagement.

H4: Hindrance stressors are negatively related to academic engagement.

H5: Academic engagement is positively related to innovative behavior.

H6: Academic engagement mediates the relationship between the challenge-hindrance stressors and innovative behavior.

## Relaxation as a moderator

Given the negative impact of hindrance stressors on academic engagement, what can medical postgraduates do to buffer the effects of these stressors on academic engagement, as well as the indirect negative impact on innovative behavior through academic engagement? According to the Conservation of Resources Theory, individuals strive to acquire, build, and protect their resources [25,35]. Demand-shielding activities can help individuals prevent loss of resources to counteract stress, taking time for relaxation as a demand-shielding activity provide them with resource recovery pathway [36]. Some empirical evidences have suggested that relaxation at work is associated with more vitality and less fatigue [37]. Furthermore, the Recovery Experience Theory posits that relaxation is characterized by a state of low activation, demanding minimal cognitive effort [38]. Operating as a tranquil cognitive state, relaxation serves to facilitate the restoration of psychological resources and the alleviation of tension [39,40]. Engaging in relaxation techniques such as mindfulness meditation, deep breathing exercises, or pursuing leisure activities unrelated to work can reduce stress levels [41,42]. By diverting attention from pressure and fostering a sense of relaxation, medical postgraduates may replenish their resources and mitigate the negative influence from hindrance stressors to their academic engagement, and in turn improve their innovative behavior.

Furthermore, the role of relaxation extends beyond simple stress management; it acts as a strategic response to stressors and varies significantly among individuals. This variability underscores its function as a moderator, individuals with higher levels of relaxation generally experience fewer negative effects from stressors than those with lower levels. This distinction highlights the importance of relaxation in moderating the relationship between stressors and academic outcomes. Meanwhile, relaxation includes a wide array of easily adoptable activities that fit seamlessly into daily routines for medical postgraduates, offering practical benefits for students in high-pressure environments [38,41]. By focusing specifically on relaxation, our study aims to provide actionable insights that can inform strategies to enhance academic engagement and foster a conducive environment for innovation. Incorporating relaxation practices into medical training programs represents a proactive approach to managing stress and maintaining high levels of engagement [36,42]. It not only mitigates the adverse effects of stressors on academic engagement but also enhances the overall well-being and innovative capacities of students, making it a cornerstone of effective educational strategies in demanding academic settings. Therefore, we speculate that:

H7: Relaxation moderates the relationship between hindrance stressors and academic engagement.

H8: The indirect effect of hindrance stressors on innovative behavior through academic engagement is moderated by relaxation.

## Method

### Participants and procedure

The participants for our survey were systematically recruited from three distinct medical universities in China, each representing a unique segment of the medical academic community. China Medical University is recognized for its high research standards and medical education, making it one of the top-tier medical universities in the country. Air Force Military Medical University offers a distinctive perspective with its military medical school background, while Hubei University of Medicine caters to regional healthcare needs, representing local medical colleges. This diverse selection was aimed at enhancing the sample’s variety and minimizing potential biases from a homogeneous study environment. The questionnaires were administered by an online survey among medical postgraduates of each college. They are asked to fill voluntarily online questionnaires and assured of confidentiality and anonymity of the provided data. A total of 1030 medical postgraduates (full-time research master’s degree) from three universities were invited to participate in this study, including a variety of medical specialties. We received 440 survey responses, resulting in a response rate of 42.7%.

To ensure the reliability and robustness of our findings, a rigorous sample size calculation was performed using a two-pronged approach. Initially, we employed the Monte Carlo simulation method, well-regarded for its precision in simulating study conditions. We set the number of simulation replications at 1,000, with model parameters based on preliminary findings, aiming for a statistical power of 0.85. This meticulous process indicated that at least 400 effective samples were required to achieve a moderated mediation effect with significant and meaningful outcomes. Additionally, we supplemented this method with traditional sample size calculations based on the number of questionnaire items, adhering to commonly accepted guidelines suggesting a 5:1 to 10:1 participant-to-item ratio [43,44]. With 36 items in our questionnaire, this guideline directed us towards a target range of 180 to 360 participants, further validating our sample size estimation and enhancing methodological rigor.

All participants were recruited in 2023 using the Chinese online survey platform Wenjuanxing (www.sojump.com). Cluster sampling was employed in this study. The questionnaire was divided into two parts: the cover page, which explained the study’s objectives, ethical considerations, and gathered demographic information including age, gender, grades, and work experiences; and the main section, which as detailed in the ‘Research Measurement,’ was meticulously designed to measure our variables. Upon completing the survey, participants were rewarded with a 10 RMB digital red envelope as compensation for their time and effort. A total of 437 responses valid for our final analysis. Among the surveyed postgraduates, 40.3% were male and 59.7% were female, with an age range between 21 and 40 years (*M* = 25.71, *SD* = 2.78). Around 42.8% of these students were first-year postgraduates, 34.3% were in their second year, and 22.9% were in their third year. Furthermore, 32.3% of the postgraduates had work experience exceeding six months.

### Measures

The questionnaires included participants’ demographic data (gender, age, grade, work experience), Challenge-Hindrance Stressors Measure, Utrecht Work Engagement Scale-Student, Innovative Behavior Measure, Recovery Experience Questionnaire. All the measures were used in Chinese version for survey (See the Appendix).

#### Challenge and Hindrance Stressors

This study assessed Challenge and Hindrance Stressors with the scale proposed by Cavanaugh et al. [16]. It’s adopted by six challenge-related items and three hindrance-related items. An example of a challenge-related item was ‘The number of projects and assignments I have’ and an example of a hindrance-related item was ‘The amount of red tape I need to go through to get my study done.’ Responses were collected using a 5-point Likert scale in which 1 was ‘never’ and 5 was ‘very often.’ In this study, the Cronbach alpha coefficient for Challenge Stressors and Hindrance Stressors was 0.85, 0.80 respectively. The CFA model fit indices are χ^2^/df = 5.09, CFI = 0.941, TLI = 0.908, SRMR = 0.067, RMSEA = 0.097.

#### Academic Engagement

Academic engagement was assessed with the 17-item UWES-S [28] that includes three subscales for using in student samples: Vigor (VI; 6 items), Dedication (DE; 5 items), and Absorption (AB; 6 items). All items are scored on a 7-point rating scale ranging from 1 (never) to 7 (always). For instance, the item ‘When I’m doing my work as a student, I feel bursting with energy.’ from the Vigor dimension; the item ‘I find my studies full of meaning and purpose.’ from the Dedication dimension; the item ‘Time flies when I am studying.’ from the Absorption dimension. In this study, the Cronbach alpha coefficient for Academic Engagement was 0.96. The CFA model fit indices are χ^2^/df = 3.97, CFI = 0.951, TLI = 0.941, SRMR = 0.037, RMSEA = 0.082.

#### Innovative Behavior Measure

The 6-item Innovative Behavior Measure is a validated tool used to measure innovative behavior, which involves both the generation and implementation of ideas [3]. An example of an innovative behavior-related item was ‘Searches out new technologies, processes, techniques, and/or product idea.’ and ‘Develops adequate plans and schedules for the implementation of new ideas.’ Participants could answer on a 5-point scale ranging from 1 ‘strongly disagree’ to 5 ‘strongly agree’. In this study, the Cronbach alpha coefficient for Innovative Behavior was 0.90. The CFA model fit indices are χ^2^/df = 3.78, CFI = 0.987, TLI = 0.972, SRMR = 0.021, RMSEA = 0.080.

#### Relaxation

We used the Relaxation Scale from the Recovery Experience Questionnaire to measure the extent to which participants take time for relaxation [38]. Participants could answer on a 5-point scale ranging from 1 ‘strongly disagree’ to 5 ‘strongly agree’. Sample items for relaxation include: ‘I use the time to relax’ and ‘I do relaxing things.’ In this study, the Cronbach alpha coefficient for Relaxation was 0.88. The CFA model fit indices are χ^2^/df = 0.02, CFI = 1.000, TLI = 1.007, SRMR = 0.001, RMSEA = 0.000.

### Common method bias test

Data from a single source such as a questionnaire survey are subject to common method bias. To effectively control common method bias, we applied methods that ensured the anonymity of questionnaires, and explained that answers were not classified as just ‘yes’ or ‘no’ so that respondents have the choice to express their real ideas [45,46]. Additionally, the questionnaire survey was administered to postgraduates in different medical universities in China. Furthermore, we referred to the single factor test method proposed by Harman [47] to test the common variance of the sample data. Exploratory factor analysis of all items in the questionnaire was conducted. The results showed that the total number of factors with eigenvalues greater than 1 was 8, and the variance of the first factor was 36.30% which was less than the critical value of 40%. Therefore, there was no obvious common method bias in the data of this study.

### Statistical analysis

The study employed descriptive and correlation analyses, utilizing SPSS version 23.0 for preliminary examination. Further analysis on the prediction capability of the structural model and the link between constructs were examined using Mplus 8.3 software. Our methodological approach included testing a mediation model, along with evaluating a moderated mediation model. Path analysis was used to investigate the specific relationships among variables and controlled for several demographic factors, including gender, age, grade, and work experience. To assess the significance of the mediation paths, we employed the bootstrap method, setting the bootstrap sample size at 5000 to generate 95% confidence intervals (CIs) for the parameter estimates. The determination of indirect effects’ significance within the bootstrapped datasets hinges on whether zero falls within the 95% confidence intervals; intervals that include zero suggest that the indirect effects are statistically non-significant [48].

### Ethical approval

Our study received ethical approval from the Hubei University of Medicine Ethics Committee (Reference No: 2022RE043). All methods were carried out in accordance with the Declaration of Helsinki and approved by the aforementioned ethics committee. Participants signed informed consents electronically to confirm their voluntary participation and their understanding of the information provided. Confidentiality and anonymity of the participants’ responses were strictly maintained throughout the study, with data being accessible only to the research team for analysis purposes.

## Results

### Preliminary analysis

Table 1 and Table 2 present the descriptive statistics for the variables and correlation matrix among them. In Table 2, gender, age, grade, and work experience are included as control variables. The results (Table 2) indicated that hindrance stressors were negatively related to academic engagement (*r* = −0.33, *p <* 0.001). Challenge stressors were found to be positively associated with innovative behavior (*r* = 0.14, *p =* 0.005), while hindrance stressors were negatively related to innovative behavior (*r* = −0.23, *p* < 0.001). There were no significant association between challenge and hindrance stressors and relaxation, but academic engagement and innovative behavior were both positively related to relaxation (*r* = 0.19, *p* < 0.001; *r* = 0.39, *p* < 0.001). Furthermore, academic engagement was significantly positively related to innovative behavior (*r* = 0.57, *p* < 0.001).

**Table 1.** Minimum, maximum, means, standard deviations, skewness, kurtosis, reliability.

Variables	Minimum	Maximum	M	SD	Skewness	Kurtosis	Reliability
1. CS	1.00	5.00	3.22	0.79	−0.16	0.07	0.85
2. HS	1.00	5.00	3.17	0.94	−0.17	−0.47	0.80
3. AE	1.00	7.00	3.89	0.92	−0.11	2.60	0.96
4. IB	1.00	5.00	3.28	0.73	−0.33	0.66	0.90
5. Relaxation	1.00	5.00	3.90	0.71	−0.74	1.78	0.88

*N* = 437. “CS” = challenge stressor, “HS” = hindrance stressor, “AE” = academic engagement, “IB” = innovative behavior.

**Table 2.** The correlation matrix among variables.

variables	1	2	3	4	5	6	7	8	9
1. gender	1								
2. age	−.18**	1							
3. grade	−.03	.42**	1						
4. work experience	.13**	−.61**	−.20**	1					
5. CS	−.05	.12*	.12*	−.14**	1				
6. HS	.11*	.02	.04	−.02	.31**	1			
7. AE	.02	.04	−.02	−.03	.05	−.33**	1		
8. IB	−.08	.06	−.02	−.12*	.14**	−.23**	.57**	1	
9. Relaxation	.06	−.08	−.04	.00	−.01	−.03	−.20**	.39**	1

*N* = 437. *p < .05, **p < .01, two-tailed. “CS” = challenge stressor, “HS” =hindrance stressor, “AE” = academic engagement, ‘IB’ = innovative behavior.

### Path analysis

#### Testing the mediating role of academic engagement

The results reported in Table 3 showed that the mediation model had good fit indices, with *χ*^*2*^(4) = 4.25, *p* = 0.37 (*χ*^*2*^*/df* = 1.06), CFI = 1.00, TLI = 1.00, RMSEA = 0.01 (90% CI = 0.00–0.09), SRMR = 0.02. After controlling for the gender, age, grade and work experience, a positive relationship was found between challenge stressors and innovative behavior in the absence of the mediator (*β* = 0.13, *p* = .016). However, the direct relationship between hindrance stressors and innovative behavior was not significant. Moreover, a positive and medium association was found between challenge stressors and academic engagement (*β* = 0.16, *p* = .007), whereas a negative and significant association was found between hindrance stressors and academic engagement (*β* = − 0.38, *p* < .001). To evaluate the significance of indirect effects, as shown in Table 3, both indirect effect of challenge stressors and hindrance stressors on innovative behavior through academic engagement were significant (effect = 0.09, 95% C.I. [0.02, 0.16]; effect = − 0.20, 95% C.I. [−0.28, −0.14]).

**Table 3.** Standardized indirect effects and 95% confidence intervals for the mediation model.

		95% CI	
Model pathways	Estimated	Lower	Upper
Challenge stressors → academic engagement → innovative engagement	−.09^a^	0.02	0.16
Hindrance stressors → academic engagement → innovative engagement	−.20^a^	−0.28	−0.14

^a^Bootstrap confidence intervals that exclude 0.

#### Moderated mediation test taking relaxation as the moderator

**Figure 1.**
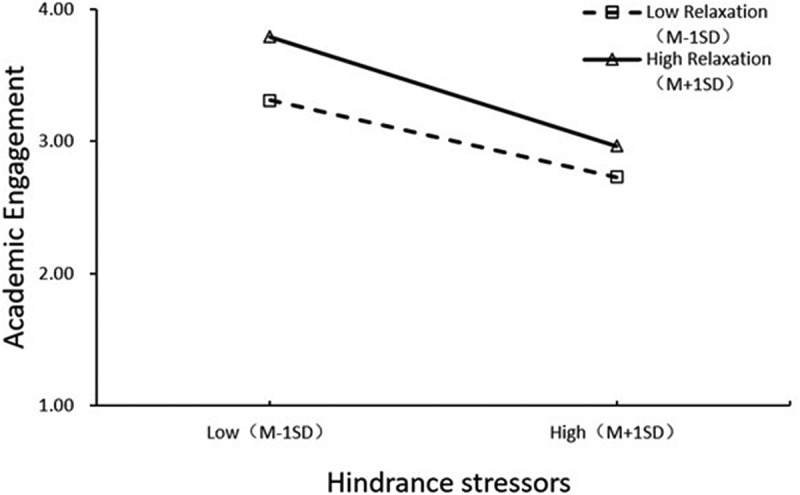
Moderation plot with relaxation as moderator.

The main results of moderated mediation analysis generated by Mplus 8.3 are presented in the Tables 4 and 5. As can be seen from the mediator variable model for predicting academic engagement, after controlling for gender, age, grade and work experience, challenge stressors were positively correlated with academic engagement (*β* = 0.14, *p* = 0.005), while hindrance stressors were negatively associated with academic engagement (*β* = −0.35, *p* < .001). The interaction term of hindrance stressors and relaxation was negatively correlated with academic engagement (*β* = −0.06, *p* = 0.029). Namely, relaxation moderated the association between hindrance stressors and academic engagement. To better understand the moderating effect of relaxation, the plot of the relation between hindrance stressors and academic engagement at two levels of relaxation (1 *SD* below the mean and 1 *SD* above the mean) was described in Figure 1. With reference to Figure 1, for individuals with high level of relaxation (1 *SD* above the mean), the negative relationship between hindrance stressors and academic engagement was attenuated. Conversely, for individuals with low level of relaxation (1 *SD* below the mean), the negative relationship between hindrance stressors and academic engagement was more pronounced. To further verify the moderated mediation relationships, we examined the indirect effect of hindrance stressors on innovative behavior via academic engagement to be different across high and low levels of relaxation. As can be seen from the dependent variable model for predicting innovative behavior, after controlling for gender, age, grade and work experience, relaxation was found to moderate the mediation of academic engagement on the relationship between hindrance stressors and innovative behavior. For individuals with high level of relaxation, the indirect effect of hindrance stressors on innovative behavior through academic engagement was weaker (*β* = −0.18, *p* < .001), whereas for individuals with low level of relaxation, the indirect effect of hindrance stressors on innovative behavior through academic engagement was stronger (*β* = −0.12, *p* < .001). The indirect effects for high and low levels of relaxation were statistically different (*β* = −0.05, *p* = 0.031). These results showed that the indirect effect of hindrance stressors on innovative behavior through academic engagement was also moderated by relaxation. Overall, this model explained 19.5% of variance in academic engagement, 35.6% of variance in innovative behavior. By contrast, relaxation wasn’t found to moderate the association between challenge stressors and academic engagement, and the indirect effect was not significant, either.

**Table 4.** Coefficient estimates for the moderated mediation Model.

	Dependent Variable = Academic Engagement				Dependent Variable = Innovative Behavior			
Variable	*β*	*SE*	*t*		*β*	*SE*	*t*	
**Control Variables**								
Gender	0.11	0.08	1.41		− 0.11	0.06	−1.83	
Age	0.02	0.02	1.05		− 0.01	0.02	−0.80	
Grade	−0.05	0.07	−0.84		−0.02	0.04	−0.48	
Work	−0.01	0.11	−0.11		−0.18*	0.08	−2.24	
**Independent Variables**								
Challenge stressors	0.14**	0.05	2.80		0.09**	0.04	2.62	
Hindrance stressors	−0.35***	0.05	−7.48		−0.07	0.03	−2.00	
**Moderator**								
Relaxation	0.18**	0.05	3.37					
**Interaction Term**								
CS× Relaxation	−0.02	0.03	−0.64					
HS× Relaxation	−0.06*	0.03	−2.18					
**Mediator**								
Academic Engagement					0.43***	0.04	11.21	
***R***^**2**^	0.195				0.356			

*N*=437. *p < .05, **p < .01, two-tailed. “CS” = challenge stressor, “HS” = hindrance stressor.

**Table 5.** Effect analysis at values of the moderator.

	*β*	Boot SE	Boot LLCI	Boot ULCI
**Conditional direct effect analysis at values of the moderator**				
Relaxation (M-1SD)	−.29***	.05	−.39	−.19
Relaxation (M+1SD)	−.41***	.06	−.53	−.30
**Conditional indirect effect analysis at values of the moderator**				
Relaxation (M-1SD)	−.12***	.02	−.17	−.08
Relaxation (M+1SD)	−.18***	.03	−.24	−.12

## Discussion

The main objective of this study was to investigate the relationship between challenge-hindrance stressors and innovative behavior among medical postgraduates in China. We also examined the mediating effect of academic engagement and the moderating role of relaxation in the relationship between two stressors and innovative behavior. Overall, our hypotheses were partially supported, representing a novel exploration in the context of Chinese medical postgraduate education, particularly using the two-dimensional stressor framework. Our findings may contribute to the understanding of how challenge-hindrance stressors relate to innovative behavior in medical higher education and provide empirical evidence for interventions aimed at enhancing the innovative behavior of medical postgraduates.

First of all, the findings of this study supported the hypotheses 1. Challenge stressors exhibited a significant positive correlation with innovative behaviors among Chinese medical postgraduates. This aligns with the notion proposed by Cavanaugh et al. [16], which suggests that challenge stressors are perceived as opportunities for growth and learning, often involving demanding tasks that offer a sense of control and mastery. In the specific context of medical postgraduates, these stressors appear to stimulate innovative behavior, echoing the findings from the occupational settings [17,49–51]. In addition, the interplay of challenge stressors with innovative behavior can also be interpreted through the lens of the Expectancy Theory [23] and the Job Demands-Control Model [24]. Challenge stressors encourage proactive strategies and a sense of mastery, leading to higher innovative outputs. Meanwhile, the Conservation of Resources Theory [25] emphasizes on the strategic investment of resources to garner greater rewards. This sheds light on why challenge stressors might lead to increased innovative behavior: medical postgraduates perceive these stressors as opportunities worth investing their time, energy, and effort in, potentially leading to innovative outcomes. Intriguingly, contrary to the findings in some work environments [19–21], we did not find a significant direct negative correlation between hindrance stressors and innovative behavior among medical postgraduates. Instead, their effect was mediated by academic engagement. Hence, our hypotheses 2 was not supported. This discrepancy might be attributed to a confluence of factors. Primarily, medical postgraduates are typically trained in high-pressure, high-risk environments, which may inherently cultivate a certain degree of resilience and adaptability. This environment may prompt them to find ways to adapt to or overcome hindrance stressors, rather than directly suppressing their innovative behavior. Additionally, innovation in medical education often arises from problem-solving in complex and uncertain scenarios. Hindrance stressors, such as resource limitations or interpersonal conflicts, while frustrating, might not directly inhibit innovative behavior but may influence it indirectly through certain mechanisms. The mediating role of academic engagement is crucial in this context. Hindrance stressors can affect the degree of academic engagement of medical postgraduates, and a lower level of engagement might lead to a reduction in innovative behavior. Therefore, the impact of hindrance stressors on innovative behavior is more indirect, channeled through their influence on academic engagement rather than exerting a direct effect on innovative behavior.

Secondly, this study has shed light on academic engagement as a potential mechanism that underlying the relationship between challenge-hindrance stressors and innovative behavior among medical postgraduates in China. Our hypothesis 3, 4, 5, and 6 were tested. The nuanced mediating role of academic engagement emerges distinctly in this context. Challenge stressors, characterized by their potential for growth and learning, seem to encourage medical postgraduates to engage more deeply in their academic endeavors. This enhanced engagement facilitates a conducive environment for innovation, as it encourages the exploration of new ideas, problem-solving, and the application of learned knowledge in novel ways [32,52]. Conversely, hindrance stressors, which are often perceived as obstacles to personal and academic development, tend to diminish this level of engagement. The pressure and frustration arising from these stressors can result in a decreased willingness to fully engage in academic activities. This not only adversely affects immediate academic performance but also limits the broader capacity for innovative thought and creativity, as students are less likely to encounter or tackle the challenges that can inspire innovative solutions [31,32]. Furthermore, the impact of academic engagement as a mediator is underscored by theories, including the Self-Determination Theory and the Conservation of Resources Theory. These theories suggest that when students perceive their academic environment as supportive and enabling (characteristic of challenge stressors), they are more likely to feel motivated and to allocate their resources towards creative and innovative efforts. Conversely, when faced with hindrance stressors, the perceived lack of support and increased obstacles may lead to resource conservation, thereby diminishing the potential for innovative behavior [33,52]. Thus, the role of academic engagement in mediating the effects of challenge and hindrance stressors on innovative behavior highlights the importance of creating educational environments that reduce hindrance stressors while fostering challenge stressors. This balance is crucial not only for maintaining high levels of academic engagement but also for promoting the innovative capabilities essential for the growth and development of medical postgraduates in China.

Thirdly, the results of our study confirmed that the direct path from hindrance stressors to academic engagement, and the indirect effect of hindrance stressors on innovative behavior through academic engagement, were moderated by relaxation. The hypotheses 7 and 8 were strongly affirmed by these findings. For medical postgraduates who engaged in higher levels of relaxation activities, the negative impact of hindrance stressors on academic engagement was significantly reduced. This is in line with the Conservation of Resources Theory [25,35], which emphasizes the importance of resource preservation and recovery in mitigating stress. Relaxation activities buffer and protect students from the depletion of psychological resources often caused by hindrance stressors [36]. Furthermore, the findings also indicated that the indirect effect of hindrance stressors on innovative behavior mediated by academic engagement, is less pronounced among those with higher levels of relaxation. This suggests that relaxation not only helps in managing the immediate negative effects of stressors on academic engagement but also plays a crucial role in maintaining the capacity for innovative behavior. According to the Recovery Experience Theory [36], relaxation aids in the restoration of mental resources and essential for innovative thinking and problem-solving. Some empirical evidences in work literature proved that taking a midday break to walk and relaxing in the park have been found to be conducive to reducing individual work-related tension [40], resulting in a decreased sense of fatigue, and even boost employee creativity and task performance [53,54]. Specifically, the positive emotions acquired through relaxation can assist individuals in thinking more openly, constructively, and creatively [55]. These beneficial effects of relaxation align well with educational settings, particularly for medical postgraduates. The challenging and often high-pressure environment of medical postgraduate studies can significantly benefit from the application of relaxation techniques. By incorporating activities such as mindfulness meditation, deep breathing exercises, or leisurely walks [37,42,56], medical postgraduates can mitigate the adverse effects of hindrance stressors on their academic engagement and innovative capacities. Such relaxation practices can provide much-needed mental reprieves, allowing students to rejuvenate and return to their academic challenges with renewed vigor and a fresh perspective. In summary, these results provide empirical support for Hypotheses 7 and 8, highlighting the pivotal role of relaxation as a moderating factor in the relationship between hindrance stressors and both academic engagement and innovative behavior. It underscores the need for strategies that foster relaxation to optimize academic engagement and preserve the innovative potential of medical postgraduates in high-stress academic settings.

In essence, this study extends the existing literature on stressors and innovative behavior by contextualizing these dynamics within the field of medical postgraduate education. It underscores the nuanced roles that different types of stressors play in influencing the innovative behaviors of individuals in high-pressure academic environments. These insights not only contribute to the theoretical understanding of stressor impacts but also have practical implications for designing educational strategies and support systems that foster innovation while managing stressors effectively.

## Limitations and future direction

Despite the relatively rich results obtained in this study, there are still some limitations that need to be addressed in future studies. Firstly, the cross-sectional design of this research limits the ability to draw causal inferences. Longitudinal or experimental designs in future studies could provide more robust evidence of the causal relationships between challenge-hindrance stressors, academic engagement, and innovative behavior among medical postgraduates. Secondly, our study relied primarily on self-reported measures, which could be subject to response biases such as social desirability or self-assessment inaccuracies. Incorporating multi-source data could enhance the reliability and validity of the findings. Thirdly, the sample was restricted to medical postgraduates in China. While this provides valuable insights into this specific demographic, the generalizability of the results to other fields of study or cultural contexts may be limited. Future research could replicate this study in different disciplines. A further limitation is that the limited examination of various stress-management strategies. Future research could more thoroughly investigate a range of recovery activities that serve to buffer the effects of stressors, thereby gaining a deeper understanding of how different resource-building and demand-shielding activities operate and their effectiveness. Meanwhile, the potential of other variables that could serve as moderators, such as personal traits, coping mechanisms, and different environmental conditions could be explored in the future research. Finally, we only investigated the mediating role of academic engagement. Future research could explore the impact of stressors on outcome variables under different mediating effects, to uncover more detailed mechanisms of how dual stressors influence innovative behavior.

## Practical implications

This study provides crucial insights for medical universities in China, especially in developing policies and practices that effectively manage stressors and foster innovative behavior among medical postgraduates. Our findings underscore that those different types of stressors have varying effects on medical postgraduates, not all stress necessarily leads to negative outcomes. Challenge stressors can even be beneficial, actively engaging medical postgraduates in their studies and research, and subsequently triggering innovative behavior. Medical universities and supervisors should carefully design their programs to include challenging yet achievable goals. These goals could involve advanced medical research projects or complex clinical tasks that encourage postgraduates to engage in innovative thinking and problem-solving. Conversely, reducing the negative impact of uncontrollable factors, such as hindrance stressors, on medical postgraduates can help students engage more fully in academic activities. Medical universities can simplify procedural processes, enhance social support, and enrich academic resources to alleviate the stress of medical postgraduates. The significant mediating role of academic engagement in this context highlights the importance of fostering deep, meaningful involvement in medical studies. Educational strategies should focus on activities that promote deep learning and meaningful engagement with academic content. This can be achieved through interactive teaching methods, collaborative projects, and practical research experiences that align with students’ interests and professional goals. Additionally, interventions aimed at enhancing engagement, such as mentorship programs, collaborative learning environments, and practical research opportunities, can be implemented. These initiatives can boost students’ motivation and interest in their academic work, thereby enhancing their innovative capabilities. Lastly, the moderating role of relaxation underscores the need for medical universities to integrate relaxation and stress management practices into their programs. Medical universities could provide resources and opportunities for relaxation, such as mindfulness training, yoga classes, or dedicated relaxation spaces. These initiatives can help students manage stress effectively, enabling them to maintain high levels of academic engagement and harness their innovative potential despite the presence of hindrance stressors. In practical terms, medical universities should consider these insights in their program design and student support services. By creating a balanced educational environment that challenges yet supports medical postgraduates, and by providing the tools to manage stress effectively, universities can significantly enhance the innovative capabilities of future medical professionals.

## Conclusion

This study probed the relationship between challenge-hindrance stressors and innovative behavior among medical postgraduates in China. Our results found that challenge stressors were positively correlated with innovative behavior, whereas hindrance stressors did not have a direct negative impact on innovative behavior. Academic engagement served as a potential mechanism that underlay the relationship between challenge-hindrance stressors and innovative behavior among Chinese medical postgraduates. Relaxation moderated the direct relationship between hindrance stressors and academic engagement, and indirect relationship between hindrance stressors and innovative behavior mediated by academic engagement. These findings could guide medical universities in designing comprehensive programs and policies that address the challenges of a demanding academic environment while promoting a culture of innovation and creativity.

## Data Availability

The data that support the findings of this study are available from the corresponding author upon reasonable request.

## Appendix Measurements

**Table:** Challenge-Hindrance Stressors Measure

Indicator	Measurement Statement
CS_1	The number of projects and/or assignments I have.
CS_2	The amount of time I spend at my study and research.
CS_3	The volume of study and research work that must be accomplished in the allotted time.
CS_4	Time pressures I experience.
CS_5	The amount of responsibility I have.
CS_6	The scope of responsibility my position entails.
HS_1	The amount of red tape I need to go through to get my research done.
HS_2	The lack of study and research security I have.
HS_3	The degree to which my study and research seem “stalled”.

**Table:** Utrecht Work Engagement Scale-Student

Indicator	Measurement Statement
VI_1	When I get up in the morning, I feel like going to class or doing research.
VI_2	When I’m doing my work as a student, I feel bursting with energy.
VI_3	As far as my studies or research are concerned, I always persevere, even when things do not go well.
VI_4	I can continue studying or doing research for very long periods at a time.
VI_5	I am very resilient, mentally, as far as my studies or research are concerned.
VI_6	I feel strong and vigorous when I’m studying or doing research.
DE_1	To me, my studies and research are challenging.
DE_2	My study and research inspire me.
DE_3	I am enthusiastic about my studies and research.
DE_4	I am proud of my studies and research.
DE_5	I find my studies and research full of meaning and purpose.
AB_1	When I am studying or doing research, I forget everything else around me.
AB_2	Time flies when I am studying or doing research.
AB_3	I get carried away when I am studying or doing research.
AB_4	It is difficult to detach myself from my studies or research.
AB_5	I am immersed in my studies or research.
AB_6	I feel happy when I am studying or doing research intensely.

**Table:** Innovative Behavior Measure

Indicator	Measurement Statement
IB_1	I always actively search out new techniques and methods in my studies and research.
IB_2	I consistently generate creative ideas in my learning and research.
IB_3	In my studies and research, I promote and champions ideas to others.
IB_4	To realize my ideas, I investigate and secure funds needed to implement new ideas.
IB_5	To achieve my ideas, I develop adequate plans and schedules for the implementation of new ideas.
IB_6	Overall, I am a person with an innovative idea in my learning and research.

**Table:** Recovery Experience Questionnaire-Relaxation scale

Indicator	Measurement Statement
RE_1	I kick back and relax.
RE_2	I do relaxing things.
RE_3	I use the time to relax.
RE_4	I take time for leisure.
